# Clinical imaging features and outcomes of intrathyroidal thymic carcinoma: an analysis of fourteen patients at a single medical institution

**DOI:** 10.3389/fonc.2025.1664612

**Published:** 2025-11-07

**Authors:** Lingling Gu, Lanfang Zhang, Wenxuan Huang, Xue Song, Xiaodong Xie, Deqin Ding

**Affiliations:** 1 Department of Medical Image Center, Jiangsu Cancer Hospital, Jiangsu Institute of Cancer Research and The Affiliated Cancer Hospital of Nanjing Medical University, Nanjing, China; 2 Department of Radiation Oncology, Jiangsu Cancer Hospital, Jiangsu Institute of Cancer Research and The Affiliated Cancer Hospital of Nanjing Medical University, Nanjing, China

**Keywords:** intrathyroidal thymic carcinoma, radiographic features, core needle biopsy, surgery, radiotherapyand chemotherapy, prognosis

## Abstract

**Objective:**

To summarize the clinical characteristics and imaging features of intrathyroid thymic carcinoma (ITTC), along with diagnostic and therapeutic approaches, to increase awareness of this rare disease.

**Methods:**

We retrospectively analyzed 14 patients with ITTC confirmed by core needle biopsy (CNB) and surgery combined with immunohistochemistry. The clinical and imaging findings, treatment, pathological findings and follow-up data of these patients were reviewed.

**Results:**

Thirteen patients were newly diagnosed and one relapsed at the original surgical site. All tumors were solitary, mostly located in the lower neck or upper chest, often in the tracheoesophageal groove with or without extension to the thyroid’s lower pole, and approximately two-thirds of patients presented with hoarseness. On CT, most lesions appeared as irregular, low-density soft-tissue masses, with calcification in two cases; contrast-enhanced CT revealed mild heterogeneous or homogeneous enhancement, and over half exhibited an arc-shaped interface with adjacent thyroid tissue. Most tumors were locally advanced, invading muscles, the supraclavicular fossa, tracheoesophageal groove, esophagus, tracheal wall, or mediastinal vessels. The diagnostic accuracy of fine-needle aspiration biopsy (FNAB) was low, whereas core needle biopsy (CNB) combined with immunohistochemistry was reliable. Ten patients underwent radical surgery, of whom three received adjuvant chemoradiotherapy and four adjuvant radiotherapy; four patients received radical chemoradiotherapy, and one received combined therapy including anlotinib, a novel tyrosine kinase inhibitor. The median follow-up was 86 months (range, 25–146), and three surgically treated patients developed local recurrence or pulmonary metastasis.

**Conclusion:**

CNB combined with immunohistochemistry is recommended when the characteristic and imaging manifestations suggest a diagnosis of ITTC. Especially for locally advanced cases, imaging-based diagnosis can be useful for analysis and to guide treatment.

## Introduction

Intrathyroidal thymic carcinoma (ITTC), also termed carcinoma showing thymus-like differentiation (CASTLE), is a rare thyroid malignancy with low-grade malignant potential, resembling thymic carcinoma histopathologically (1). Thymic tissue arises from the third and fourth branchial pouches during embryogenesis and descends into the mediastinum. ITTC, likely originating from ectopic thymic tissue within or adjacent to the thyroid or from embryonic thymic remnants, was first described by Miyauchi in 1985 as “intrathyroidal epithelial thymoma” (2, 3). Chan and Rosai renamed it CASTLE and outlined its clinicopathological characteristics (4). In 2004, the World Health Organization (WHO) recognized ITTC as a distinct thyroid tumor entity in its Classification of Thyroid Tumors (5), and in the 2017 WHO Classification of Endocrine Tumors, it was formally designated as “intrathyroidal thymic carcinoma” (6). Most reported cases occur in Asia, particularly in China and Japan (6).

Although ITTC and thymic carcinoma share similar histopathological and immunohistochemical profiles, differences exist in EGFR and p53 expression. ITTC exhibits chromosomal imbalances resembling those of thymic carcinoma (7). In addition, TERT promoter C228T mutations were reported in 22% of ITTC cases, but not in thymic carcinoma (8). Clinically, ITTC tends to affect younger patients and generally carries a more favorable prognosis than thymic carcinoma, although aggressive cases have also been documented (8, 9).

Given the rarity of ITTC, most reported cases to date consist of case reports and literature reviews (10–12), with no established guidelines for diagnosis or treatment. To enhance understanding of this disease, this study analyzed clinical and imaging data from 14 patients, summarizing their clinical characteristics, radiographic features, diagnostic process, and treatment outcomes.

## Materials and methods

### Clinical data

Fourteen ITTC patients who underwent initial surgery or biopsy at Jiangsu Cancer Hospital from February 2014 to March 2023 were retrospectively analyzed. They included 9 males and 5 females, ranging in age from 41–76 years. The recorded data included clinical characteristics, CT, MRI and PET–CT imaging features, as well as diagnosis, treatment, pathology and follow-up data. This study was approved by our hospital ethics committee, and the patients signed the informed consent form.

### Imaging protocol

#### CT technique

CT was performed using a 64-slice spiral CT system (GE Healthcare, Revolution, USA) using a high-pressure injector. Fourteen patients underwent plain scans and contrast-enhanced CT of the neck and chest in the axial plane. Patients were placed in a supine position with scans ranging from the skull base to the diaphragmatic level. The scan parameters were as follows: 120 kV tube voltage, 300 mAs tube current, pitch 0.984, matrix 512×512, slice thickness 5 mm, gantry rotation time 0.8 s, and field of view 45×45 cm. Enhanced images were obtained via intravenous injection of a nonionic contrast agent (1.2–1.5 mL/kg) (Lodixanol, Yangtze River Pharmaceutical Co., China) at a rate of 2.5 mL/s using an automatic power injector, followed by a 10 mL flush of saline solution. The axial images were reconstructed with contiguous sections of 1.25 mm slices.

### Imaging analysis

Two experienced radiologists (30 and 12 years of thyroid imaging experience), blinded to the biopsy results, retrospectively reviewed all CT scans. Discrepancies were resolved by consensus. Imaging features evaluated included tumor location, shape, diameter, margin, and radiographic characteristics (plain and enhanced features, density, calcification, invasion of surrounding structures, and lymph node metastasis). Tumor shape was classified as round/oval or irregular, and margins were categorized as well-defined or ill-defined.

### Pathological examination

A histopathological examination of surgical samples was performed via standard hematoxylin and eosin (HE) staining in conjunction with specific immunohistochemical techniques, and the main immunohistochemical markers, such as CD5, CD117, p63, thyroid transcription factor-1 (TTF-1), CK5/6, calcitonin (CT), and thyroglobulin (TG), were detected.

## Results

### Patient characteristics

This study included 14 patients, comprising 9 males and 5 females, with a median age of 53 years (range, 41–76 years).Thirteen patients presented with primary unilateral solitary tumors. One patient had recurrent disease that developed three years after inappropriate ^131^I therapy, following a prior operation for misdiagnosed thyroid carcinoma.

Patients presented with several major symptoms. Hoarseness was observed in 9 patients: 3 with concurrent neck masses, 5 isolated, and 1 accompanied by severe chest pain and asthma. Other presentations included painless, slow-growing neck masses (n=4) and a painful neck mass (n=1). None of the patients reported dyspnea or dysphagia. All the patients with hoarseness showed no abnormalities in gastroscopy and laryngoscopy, and chest CT examination showed no abnormal nodules or masses in the lungs of all patients.

Parathyroid hormone levels were decreased in 2 patients and elevated in 1. Free thyroxine levels were increased in 2 and decreased in 1 patient, while thyrotropin levels were decreased in 2 and increased in 1 patient. Serum thyroglobulin was decreased in 2 patients.

### Pretreatment diagnosis

The pretreatment clinical diagnosis was established mainly on ultrasonography and CT findings. Among the 14 patients, 9 were clinically considered to have malignant disease according to CT findings, including thyroid malignancy in 7, metastasis with unknown primary focal in 1, and thyroid carcinoma recurrence in 1. The remaining 5 patients were categorized by TI-RADS grading, including 1 case of TI-RADS 3, 1 of TI-RADS 4A, 1 of TI-RADS 4B, and 2 of TI-RADS 5.

Pathological evaluation was then performed. Three patients underwent CNB guided by ultrasonography and had pathologically confirmed ITTC. Five patients underwent preoperative FNAB guided by ultrasonography: one was diagnosed with undifferentiated carcinoma, two with poorly differentiated carcinoma, one with poorly differentiated squamous cell carcinoma, and one showed no tumor cells. Detailed clinical information is presented in **Table 1**.

**Table 1:** General information of 14 ITTC patients.

Patient No.	Age/sex	Symptom	Cytological findings	Details of operation	Lymph node metastasis	Postoperative radiotherapy	Follow up and outcome
1	56/M	hoarseness	puncture pathology showed no tumor cells	left tracheoesophageal groove mass dissection	negative	local radiotherapy + chemotherapy	120 months; dead
2	53/M	hoarseness	ITTC	cytological findings	negative	radical radiotherapy and chemotherapy	96 months; alive with NED
3	67/M	hoarseness	undifferentiated carcinoma	bilateral total thyroidectomy + central neck dissection, left ipsilateral radical neck dissection	negative	local radiation	63 months; alive with NED
4	76/F	cervical mass by physical examination	poorly differentiated carcinoma	bilateral total thyroidectomy + central neck dissection + tracheal reconstruction	positive	local radiation	25 months; pulmonary metastasis, dead
5	43/F	cervical mass by physical examination, neck pain	—	subtotal thyroidectomy	negative	local radiation	70 months; alive with NED
6	41/F	hoarseness	—	recurrent tumor resection of the esophageal muscularis + tracheal 1-5 sleeve resection	negative	local radiation	146 months; alive with pulmonary metastasis
7		hoarseness; cervical mass	—	bilateral total thyroidectomy + central neck dissection	negative	—	Lost to follow up
8	53/F	cervical mass by physical examination, painless	—	bilateral total thyroidectomy + central neck dissection	negative	local radiotherapy + chemotherapy	48 months; alive with NED
9	48/F	cervical mass, hoarseness	—	left thyroid lobectomy, left central neck dissection	negative	local radiotherapy + chemotherapy	103 months; alive with NED
10	51/M	cervical mass by physical examination, painless	low-grade squamous cell carcinoma	bilateral total thyroidectomy + central neck dissection	negative	—	113 months; alive with NED
11	59/M	cervical mass, hoarseness	poorly differentiated carcinoma	bilateral total thyroidectomy + central neck dissection; left ipsilateral radical neck dissection	negative	—	19 months; alive with recurrence; pulmonary metastasis
12	43/M	hoarseness	—	right tracheoesophageal groove mass biopsy	negative	radical radiotherapy and chemotherapy	86 months; alive with NED
13	69/M	cervical mass by physical examination, painless	ITTC	cytological findings	negative	radical radiotherapy and chemotherapy; oral antirotinib	41 months; alive with NED
14	73/M	hoarsenes; chest pain, asthma	ITTC	cytological findings	negative	radical radiotherapy and chemotherapy	33 months; alive with NED

F, female; M, male; NED, no evidence of disease.

### Imaging features

The CT imaging findings of the 14 patients with ITTC included in our study are summarized in **Table 2**.

**Table 2:** CT features of intrathyroidal thymic carcinoma and TNM staging based on imaging.

Patient No.	Tumor location/size (cm)	Shape	Margin	Enhancement	Adhesion to or invasion of trachea and/or esophageal	cTNM
1	left tracheoesophageal groove/2.2 cm	oval	well-defined	mild homogeneous enhancement	negative	cT2N0M0
2	right supraclavicular fossa, right tracheoesophageal groove and right lower pole of the thyroid /5.8 cm	irregular	ill-defined	moderate inhomogeneous enhancement	invaded the trachea and esophagus, tracheal stenosis	cT4BN0M0
3	left tracheoesophageal groove and left lower pole of the thyroid /3.9 cm	irregular	ill-defined	mild inhomogeneous enhancement	invaded the esophagus; adhered to the trachea	cT4AN0M0
4	right supraclavicular fossa, right tracheoesophageal groove and right lower pole of the thyroid /5.1 cm	oval	ill-defined	moderate homogeneous enhancement	positive invaded the trachea, tracheal stenosis	cT4AN1aM0
5	left lower pole of the thyroid /1.5 cm	irregular	ill-defined	mild homogeneous enhancement	negative	cT3BN0M0
6	left tracheoesophageal groove/2.3 cm	irregular	ill-defined	mild homogeneous enhancement	positive invaded the trachea and esophagus, tracheal stenosis	cT4AN0M0
7	left tracheoesophageal groove and left lower pole of the thyroid /2.1 cm	irregular	ill-defined	mild homogeneous enhancement	positive, invaded the trachea and esophagus	cT4AN0M0
8	left lower pole of the thyroid gland/1.5 cm	round	well-defined	mild homogeneous enhancement	negative	cT1N0M0
9	left tracheoesophageal groove and right lower pole of the thyroid gland/2.4 cm	round	well-defined	mild homogeneous enhancement	–	cT3BN0M0
10	right thyroid/4 cm	irregular	ill-defined/	mild inhomogeneous enhancement	negative	cT3BN0M0
11	left tracheoesophageal groove and left lower pole of the thyroid gland/3.1 cm	irregular	ill-defined	mild inhomogeneous enhancement,	adhered to the trachea and esophagus	cT4AN0M0
12	right lower pole of the thyroid and right suprasternal fossa/3.3 cm	irregular	ill-defined	mild inhomogeneous enhancement	adhered to the trachea	cT4BN1aM0
13	left lower pole of the thyroid, suprasternal fossa and anterior superior mediastinum/5 cm	irregular	ill-defined	moderate inhomogeneous enhancement	adhered to trachea and esophagus	cT4BN1aM0
14	right lower pole of the thyroid, tracheoesophageal groove, right anterior superior mediastinum/4.1 cm	irregular	ill-defined	mild homogeneous enhancement	positive invaded the trachea and esophagus, tracheal stenosis	cT4BN1aM0

ITTC patients was classified by the 8^th^ edition of the American Joint Committee on Cancer (AJCC) staging system for thyroid.

Tumors were single lesions that were commonly located in the lower neck and upper chest between the inferior pole of the thyroid and the anterior superior mediastinal space. Overall, 9 tumors were left-sided and 5 were right-sided.

Tumor size ranged from 1.5 to 5.8 cm, with a mean diameter of 3.3 cm. Nine primary lesions and 1 recurrent mass were irregular in shape, 2 lesions were oval, and the remaining 2 were round. Most tumors were ill-defined, and only 3 were well-defined due to their small size and clear margins.

**Figure 1:**
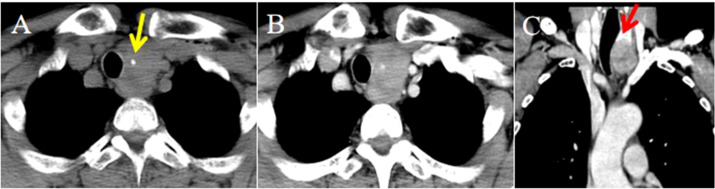
CT imaging of the ITTC. **(A)** Plain CT scan showed an irregular mass at the left tracheoesophageal groove with extension to the thyroid’s lower pole, with central calcification (yellow arrow); **(B)** After the injection of contrast medium, it exhibited slight to mild heterogeneous enhancement with small central hypodense areas; **(C)** Compared with uninvolved thyroid tissue, the tumor-thyroid interface forming an arc-shaped boundary at multiplanar reconstruction (red arrow).

**Figure 2:**
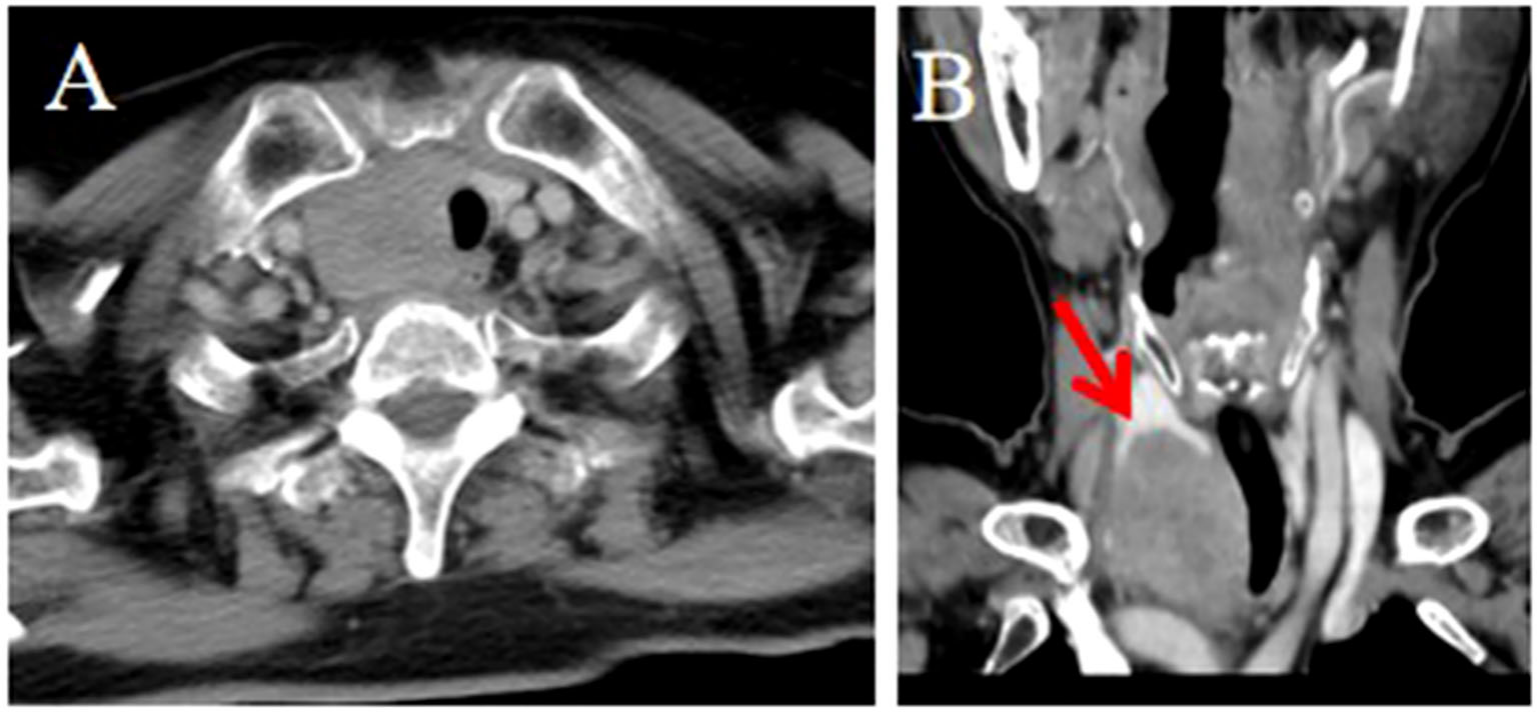
CT imaging of the ITTC. **(A, B)** Mass demonstrating homogeneous enhancement at the right tracheoesophageal groove with extension to the thyroid’s lower pole; **(B)** The tumor–thyroid interface formed an arc-shaped boundary (red arrow) at multiplanar reconstruction, involved the esophagus, causing tracheal stenosis, which was confirmed by surgery.

The lesions commonly exhibited low soft tissue density on plain CT scans with uniform attenuation, except for 5 with non-homogeneous density. The attenuation was comparable to that of adjacent muscles but significantly lower than that of the thyroid. Central calcification was observed in 2 patients (**Figure 1**). After contrast administration, 7 lesions showed mild homogeneous enhancement (**Figure 2**), whereas 4 exhibited slight to mild heterogeneous enhancement with small central hypodense areas (**Figure 1**). One lesion demonstrated moderate homogeneous enhancement, and 2 showed moderate heterogeneous enhancement.

No tumor invaded the contralateral thyroid gland. Enlarged lymph nodes were found in 4 patients at levels VI and VII according to the AJCC classification. Ten tumors presented with locally advanced disease, often invading the region between the thyroid lower pole and the anterior superior mediastinum, including adjacent muscles, the supraclavicular fossa, tracheoesophageal groove, esophagus, trachea, and occasionally mediastinal vessels. Compared with uninvolved thyroid tissue, the tumor–thyroid interface appeared relatively well demarcated in 9 patients (64.3%), forming an arc-shaped boundary (**Figures 1**, **2**).

**Figure 3:**
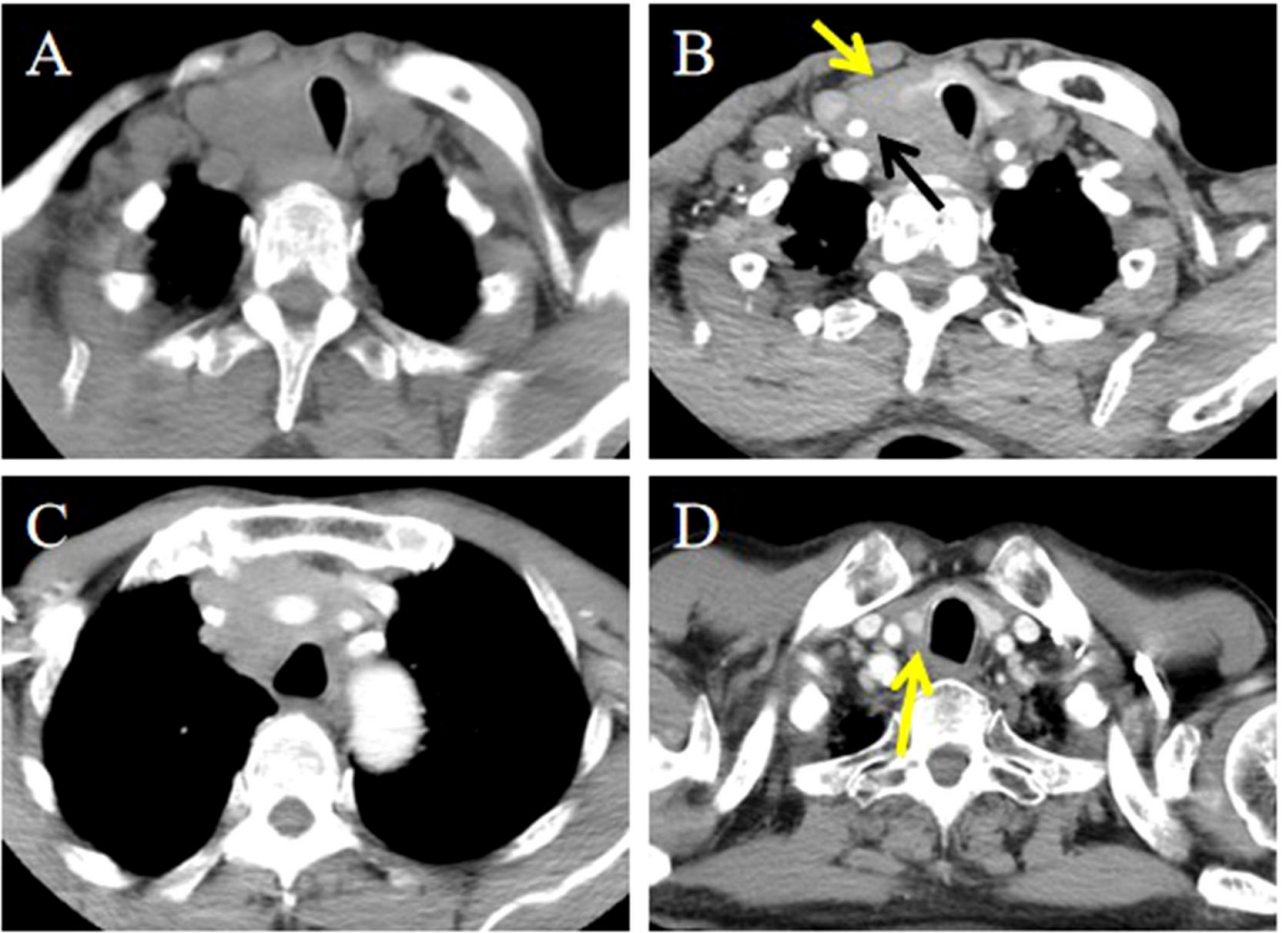
CT imaging of the ITTC before and after treatment. **(A)** Mass exhibited low soft tissue density on plain CT scans with uniform attenuation; **(B, C)** Mass demonstrated perfusion-like growth filling the vascular space, infringed strap muscle (yellow arrow); and encased the right common carotid artery (black arrow); **(D)** After completion of radiotherapy and chemotherapy, imaging modality demonstrated complete remission (yellow arrow).

**Figure 4:**
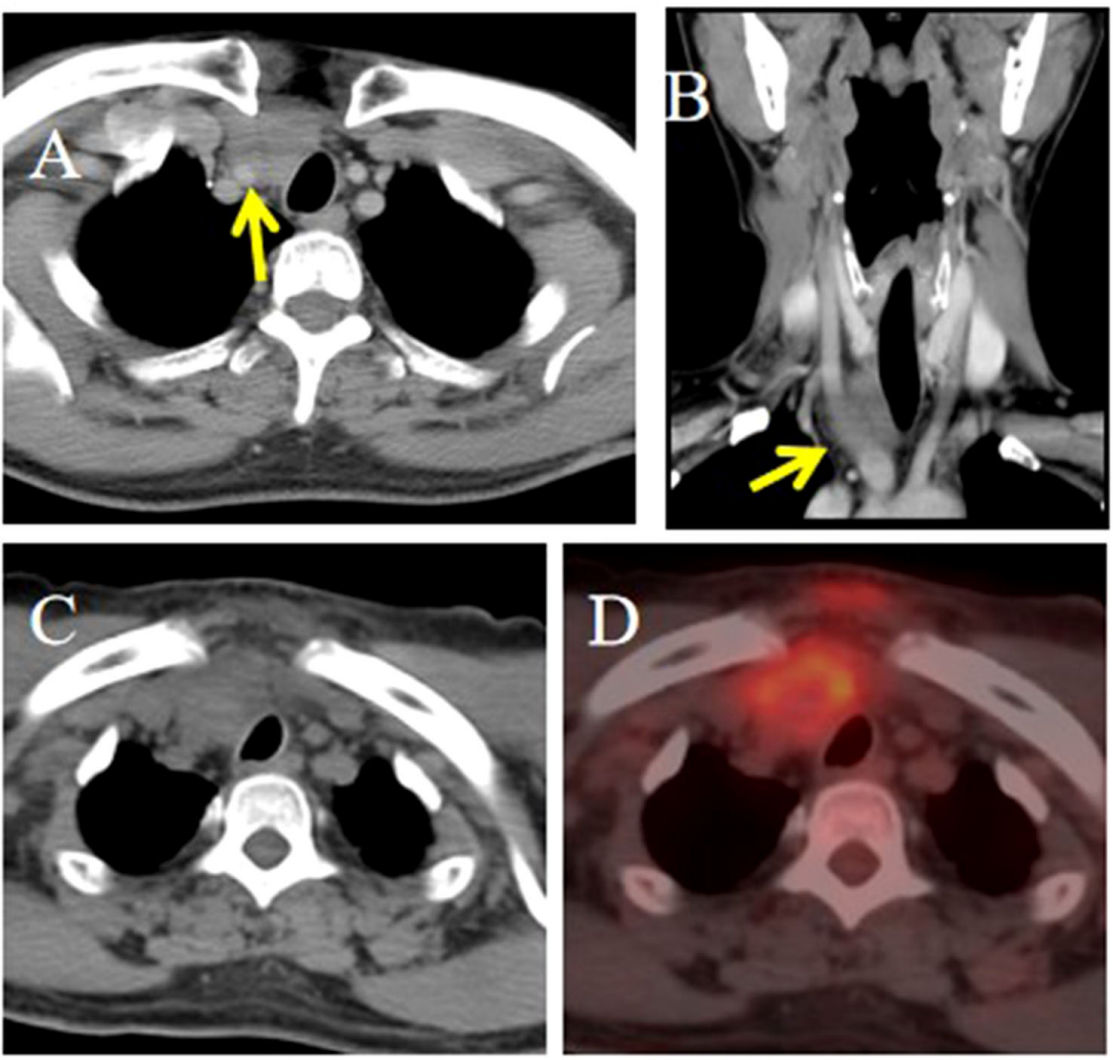
CT and PET/CT imaging of the ITTC. **(A, B)** Tumor located in the right lower pole of the thyroid and right suprasternal fossa; the tumor was inseparable from the right common carotid artery at multiplanar reconstruction (yellow arrow). **(C, D)** It demonstrated obvious focal FDG tumor uptake.

**Figure 5:**
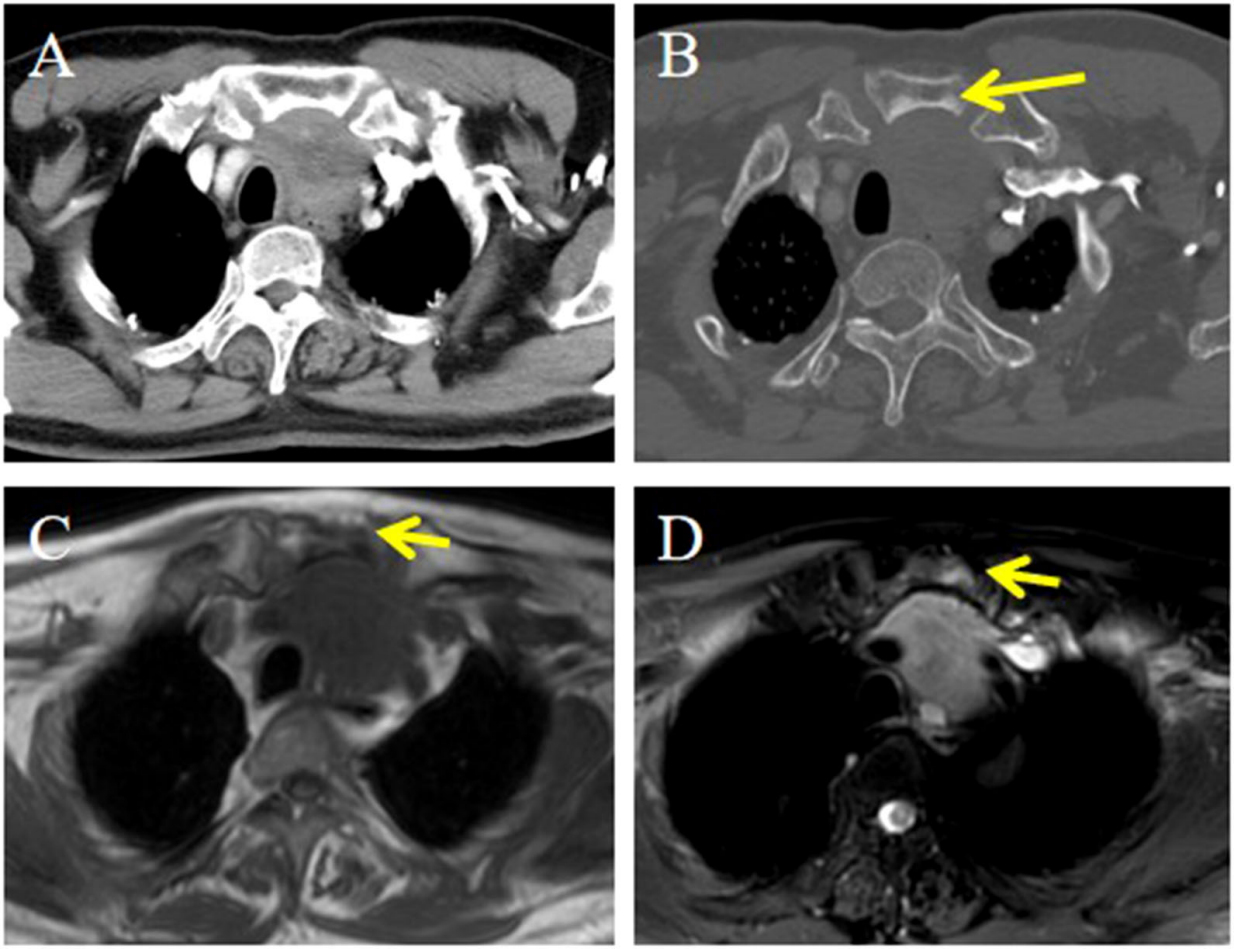
CT and MRI imaging of the ITTC. **(A)** Mass demonstrating heterogeneous enhancement at the left lower pole of the thyroid extending retrosternally into the upper mediastinum; **(B)** It showed invasion into the sternum, with heterogeneous density on CT (yellow arrow); **(C, D)** Invasive mass presented as hypointensity on axial T1-weighted imaging and slightly hyperintense on axial T2-weighted fat-suppressed sequence, invading to adjacent sternum resulting in abnormal bone signal (yellow arrow).

Strap muscle invasion occurred in 7 patients, characterized by direct tumor encasement or obliteration of the intervening fat space (**Figure 3**). The tumor involved or adhered to the trachea and esophagus in several cases, leading to tracheal stenosis in 4 patients (28.6%), with intraluminal extension into the tracheal wall or membrane (**Figures 1**, **2**). Large mediastinal vessels were invaded or encased in 4 patients (**Figures 3**, **4**). One patient demonstrated perfusion-like growth filling the vascular space (**Figure 3**). Another case showed invasion into the sternum, with heterogeneous density on CT and abnormal bone signals on MRI (**Figure 5**). In one patient, the tumor was inseparable from the right common carotid artery during intraoperative biopsy. Subsequent fluorine-18 fluorodeoxyglucose positron emission tomography/computed tomography (18F-FDG PET/CT) revealed avid focal uptake (**Figure 4**).

### Treatment and follow-up

As described in **Table 1**, 10 patients were treated with curative surgery for tumors, the most common surgical method was total thyroidectomy (6/10), included bilateral central compartment dissection (n=4), central compartment dissection and ipsilateral radical neck dissection (n=2), other types included lobectomy with central compartment dissection, subtotal thyroidectomy, trachea groove mass dissection, recurrent tumor dissection. Among the patients, 3 accepted postoperative radiotherapy (50.4–64Gy/28–32 fractions) and chemotherapy (docetaxel and cisplatin), and 4 received postoperative radiotherapy (56–64Gy/28–32 fractions).

One patient with severe tracheal stenosis underwent radical chemoradiotherapy (70 Gy/32 fractions with docetaxel and cisplatin). Similarly, three patients with extensive, unresectable tumors involving mediastinal vessels received radical chemoradiotherapy (60–64 Gy/30–32 fractions with docetaxel plus nedaplatin or albumin-bound paclitaxel and nedaplatin). One patient exhibited a suboptimal response to chemoradiotherapy and was subsequently treated with oral anlotinib, a small-molecule multitarget tyrosine kinase inhibitor (TKI).

**Figure 6:**
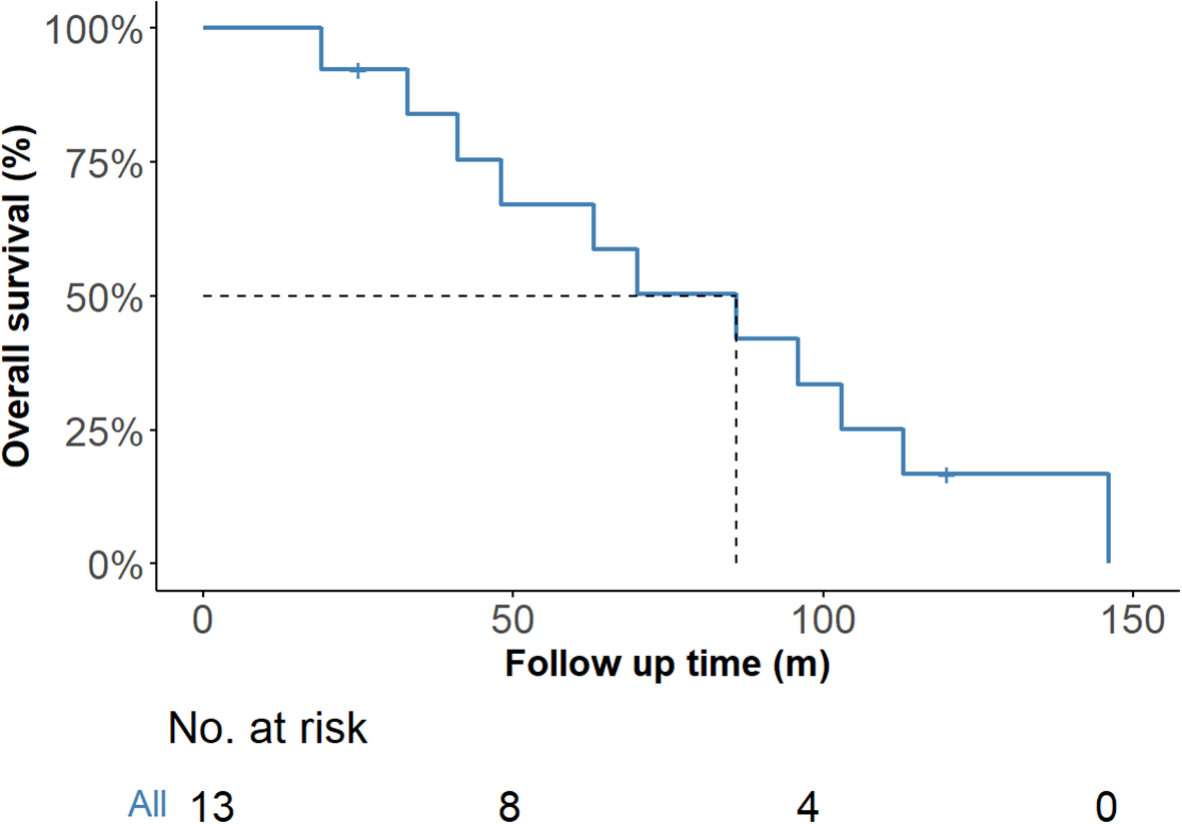
Overall survival of the 14 ITTC cases according to Kaplan–Meier. ITTC, intrathyroidal thymic carcinoma.

One patient who underwent bilateral total thyroidectomy was lost to follow-up at 3 months. Follow-up information was available for the remaining 13 patients, with durations ranging from 25 to 146 months. One patient died 120 months after the initial surgery combined with chemoradiotherapy. Local recurrence and pulmonary metastasis were detected in one patient 25 months after the initial treatment, after which this patient died. One patient underwent surgery for recurrence in the central region and received adjuvant radiotherapy after surgery, and lung metastases were observed after 146 months. One patient experienced recurrence and pulmonary metastasis 19 months after radical thyroidectomy. Nine other patients were disease free at the time of the last follow-up. **Figure 6** shows the Kaplan–Meier curve for overall survival probability, and the median OS was 86 months.

### Pathological findings

**Figure 7:**
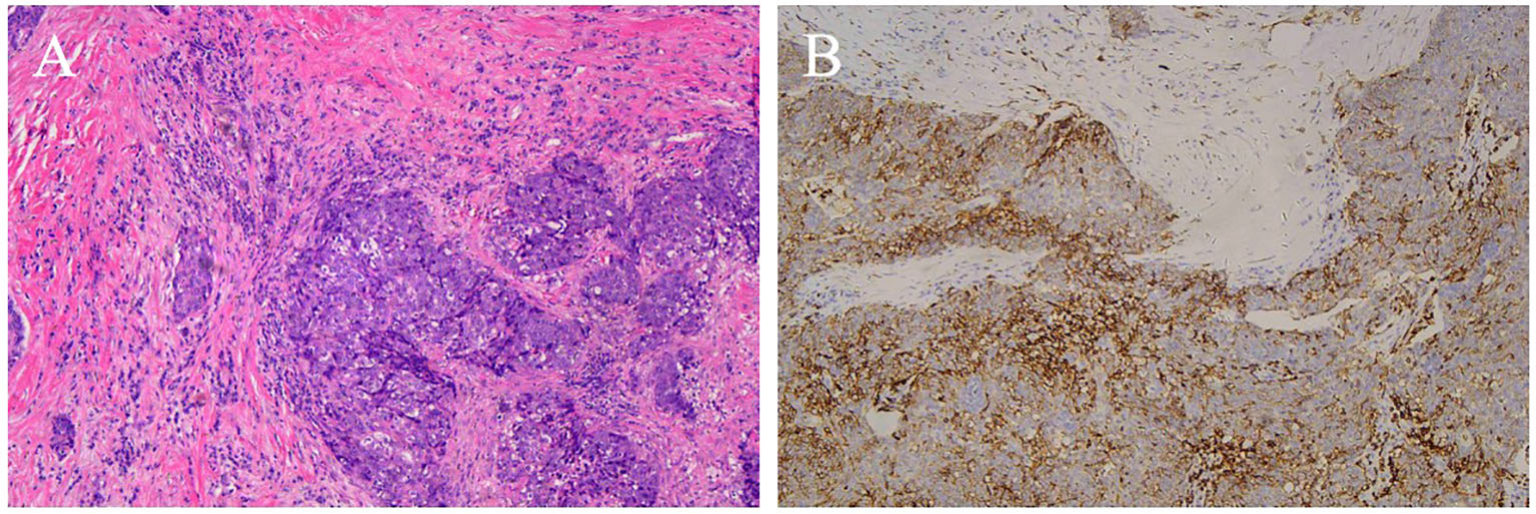
CD5 positive ITTC. **(A)** Tumor cells are arranged in irregular nests and separated by bands of dense fibrous stroma. The epithelial cells and fibrous stroma were infiltrated by amounts of lymphocytes and plasmocytes (H&E, original magnification 100×). **(B)** Neoplastic cells show immunohistochemical reactivity for CD5.

None of the surgically treated patients received a definitive diagnosis on intraoperative frozen section. Grossly, the tumors were solid, grayish-white masses without a capsule. Microscopically, they consisted of nests of epithelial cells resembling squamous cell carcinoma, arranged in sheets or islands, separated by bands of dense fibrous stroma. The epithelial cells and fibrous stroma were infiltrated by different numbers of small lymphocytes and plasmocytes. The tumor cells displayed a polygonal, or syncytial-like appearance (**Figure 7**). Tumor cells showed oval nuclei with vesicular chromatin and small distinct nucleoli. Lymph node metastasis was confirmed in one patient.

**Figure 8:**
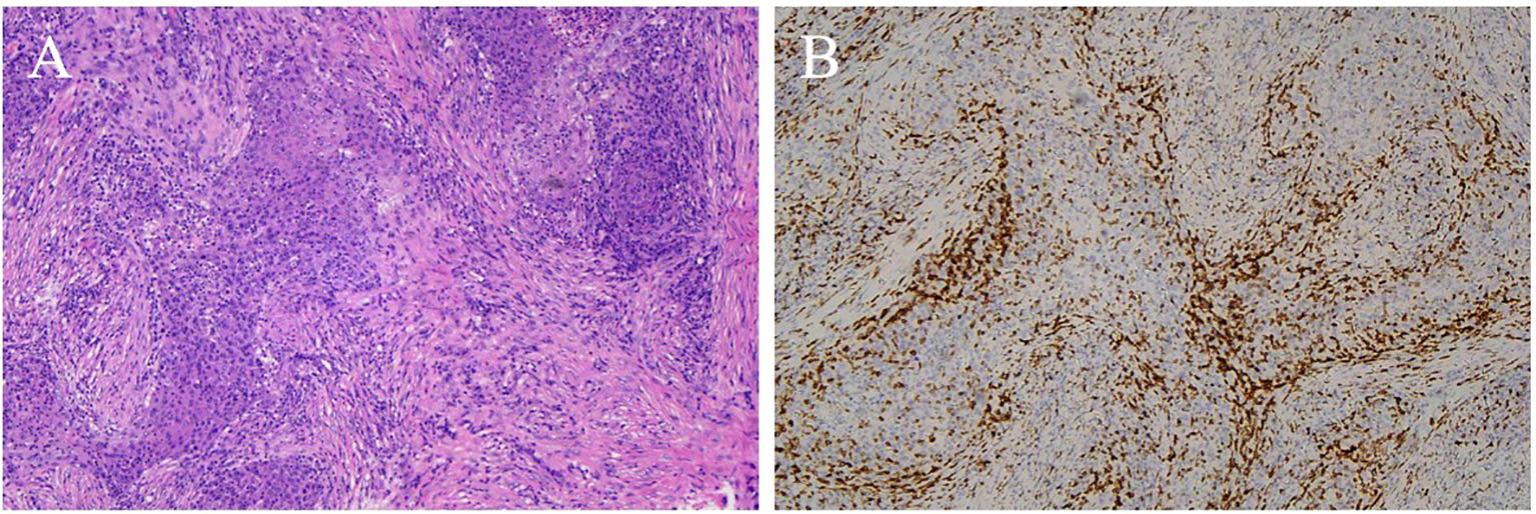
CD5 negative ITTC. **(A)** Islands of tumor cells separated by fibrous bands displaying rich of of lymphocytes and plasma cells of a carcinoma showing thymus-like differentiation of the thyroid gland, and the tumour showed syncytial-like appearance and the pushing margins with pleomorphic vesicular nuclei (H&E, original magnification 100×). **(B)** Neoplastic cells showed negative for CD5.

Immunohistochemical results were available for 13 patients. CD5 (**Figure 7**) was positive in 11 (84.6%) patients and negative in 2. Notably, the remaining 2 cases of ITTC without immunoreactivity to CD5 were positive for CK5/6 and p63 but negative for TTF-1, and on microscopically the tumour showed syncytial-like appearance and the pushing margins, fibrous septa and lymphocytic infiltrate of a carcinoma showing thymus-like differentiation of the thyroid gland, differentiated from primary thyroid squamous cell carcinoma (**Figure 8**). All tumors tested were positive for CK5/6 (10/10) and p63 (8/8), whereas all were negative for TTF-1 (9/9) and TG (6/6). CD117 expression was observed in 8 of 11 (72.7%) patients. Synaptophysin (Syn) staining was performed in 6 patients, of whom 5 showed positivity, with scattered positive cells in 3 cases.

## Discussion

ITTC tumors are categorized as thyroid carcinomas, although they resemble thymic carcinomas and are thought to originate from the intrathyroidal ectopic thymus or embryonic cervical descent of the thymus. Owing to their rarity, most masses are diagnosed as thyroid cancer or thyroid malignancy on the basis of radiographic findings. In our study, 85.7% (12/14) of cases were initially classified as thyroid cancer or thyroid malignancy radiographically. Thus, surgery was adopted as the initial treatment in 8 patients (57.1%, 8/14) owing to frequent misdiagnosis, and the definitive diagnosis relied on surgical pathology, particularly immunohistochemistry. Accurate pretreatment diagnosis is crucial, but whether surgery should be the initial choice, particularly for advanced cases, and whether clinical and imaging findings improve diagnostic accuracy remain key issues.

ITCC tumors occur in middle-aged individuals with a mean age of 50 years (12). In our study, the median age at diagnosis was 53 years, with a male-to-female ratio of 1.8:1 (9:5). Although males appeared more frequently affected in our cohort, this likely reflects the limited sample size rather than a true sex bias. Conversely, Gao observed a slight female predominance in a larger series of 89 patients (13). Thyroid hormone levels were nonspecific in our cases. Hoarseness was a prominent symptom among patients, as approximately two-third of them (64.3%) experienced it.

In our study, more than half of the tumors (64.3%) were located in the tracheoesophageal groove, with or without extension to the lower thyroid pole, often accompanied by hoarseness due to recurrent laryngeal nerve paralysis. A few tumors extended into the supraclavicular fossa, suprasternal fossa, or even the vascular space of the anterior superior mediastinum. These locations are consistent with the embryonic origin of thymic remnants.We hypothesize that the location of the tracheoesophageal groove and the resulting concomitant symptoms may be key points for distinguishing this tumor from other thyroid tumors.

Radiographically, most of the masses were irregular and ill-defined and had low soft tissue density with uniform attenuation on plain CT. Central calcification was present in 2 patients; calcification within masses has been reported in a few studies (14). On contrast-enhanced CT, tumors demonstrated mild heterogeneous or homogeneous enhancement, while marked heterogeneous enhancement was rare but has been reported (15). Importantly, neither calcification nor marked enhancement should exclude ITTC from consideration.

ITTC tumors usually present with a slow course and are prone to invading surrounding organs. The relatively small masses appeared to be round or oval in shape and confined to the tracheoesophageal groove or lower pole of the thyroid. Larger masses with irregular shapes often present locally advanced disease, grow along the lower thyroid gland or the tracheoesophageal groove, invading surrounding structures such as the supraclavicular fossa, ipsilateral strap muscles, trachea and esophagus, and suprasternal fossa, and even infiltrate the upper mediastinum vascular space. The growth characteristics in our cases were similar to those reported previously (15). Tracheal invasion leading to stenosis was seen in 28.6% of our patients, slightly lower than the 38% reported in the literature (1).

A distinctive imaging feature in our series was a well-defined margin between involved and uninvolved thyroid tissue, forming an arc-shaped boundary in over half of the cases. Best visualized on multiplanar reconstruction, this feature distinguishes ITTC from primary thyroid malignancies and has not been previously reported. It may reflect the ectopic thymic origin of ITTC rather than a thyroidal origin, though validation in larger cohorts is warranted. MRI provided no diagnostic advantage but was more sensitive in detecting extrathyroidal invasion. FDG PET/CT revealed avid focal uptake, consistent with prior reports (16).

In our study, nodal metastasis was pathologically confirmed in only one patient, and the overall incidence of nodal enlargement was low. The pattern of Level VI and VII involvement resembled that of other thyroid malignancies. Previous reports indicated that 84.3% of metastases occur in the central compartment and 15.6% in the lateral compartment, and nodal involvement is considered a prognostic risk factor (13). Ito (17) further demonstrated that larger tumor size (>4 cm) was significantly associated with nodal metastasis. During follow-up, three patients developed lung metastases, consistent with the lung being the most common metastatic site in earlier studies (18).

A definitive diagnosis of ITTC before surgery remains challenging. In our cohort, 5 patients underwent fine-needle aspiration cytology (FNAC) prior to treatment, but none yielded a definitive diagnosis. In contrast, 3 of 8 patients (37.5%) who underwent core needle biopsy (CNB) were correctly diagnosed with ITTC on the basis of pathology and immunohistochemistry. Preoperative FNAB failed to establish a diagnosis of ITTC in any patient, as the thin needles yield only cellular material rather than tissue cores. Although FNAB identified 98.5% of cases as malignant, its sensitivity for correctly diagnosing ITTC was only 8.3% (13). By contrast, CNB markedly reduces diagnostic uncertainty, offers superior accuracy, and is not associated with significant complications (19).

Regarding immunohistochemistry, most ITTC cases were positive for CD5, a marker for carcinoma of thymic origin, as well as for p63, CD117, CK5/6 and negative for thyroid transcription factor 1 (TTF-1), to distinguish it from other thyroid tumors, also described in the literature (20). Two patients with negative CD5 expression were positive for CK5/6 and p63 but negative for TTF-1, presented with typical carcinoma showing thymus-like differentiation of the thyroid gland morphologically. Expression of synaptophysin (Syn) was evaluated in a subset of patients. According to Yamazaki et al, the expression of neuroendocrine markers such as Syn and CgA in ITTC also supports the idea that this thyroid cancer originates in the thymus, as neuroendocrine markers have been reported to have a focal or dispersed positive distribution in thymus cancer (21).

The most common differential diagnosis for ITTC might involve primary thyroid malignancies. ITTC tumors confined to the lower pole of the thyroid should be distinguished from papillary thyroid carcinoma, which more often shows fine granular calcifications. Additional imaging signs include irregular rings, marginal defects, and blurred enhancement (22). Papillary carcinoma also has a high incidence of nodal metastasis, and characteristic nodes with abundant vascularity, calcification, or cystic change are useful in the differential diagnosis (23). In clinical practice, papillary carcinoma is often readily diagnosed intraoperatively, whereas in our cohort none of the ITTC patients who underwent surgery achieved a definitive diagnosis on frozen section.

Locally advanced ITTC should also be differentiated from undifferentiated thyroid carcinoma and primary squamous cell carcinoma of the thyroid. Undifferentiated carcinoma typically exhibits the hallmarks of highly aggressive disease, with nodal metastases being common. Primary squamous cell carcinoma usually affects older men, progresses rapidly, and appears as thyroid enlargement with low-density shadows, calcification, and necrosis on CT (24). Beyond thyroid primaries, ITTC must further be distinguished from isolated recurrent laryngeal nerve node metastasis of esophageal or subpharyngeal squamous cell carcinoma, which can similarly present with hoarseness. In clinical practice, when patients present with hoarseness, gastroscopy and laryngoscopy are necessary. On the basis of these considerations, when ITTC is suspected based on imaging and clinical manifestations, CNB combined with cytology and immunohistochemistry should be recommended before surgery.

The prognosis for ITTC is generally favorable, with reported 5- and 10-year survival rates of 90% and 82%, respectively (1). Gurizzan demonstrated that advanced local stage is the strongest prognostic factor for mortality (18). In our study, 64.2% (9/14) of patients were staged as T4 due to invasion of adjacent structures, necessitating extensive resection. One patient was diagnosed with poorly differentiated thyroid carcinoma by FNAB and underwent total thyroidectomy, lymph node dissection, tracheal reconstruction, and postoperative radiotherapy, but died after 25 months. Another patient, initially misdiagnosed and treated inappropriately with ^131^I, later developed local recurrence and underwent re-resection with tracheal reconstruction followed by radiotherapy, remaining alive with pulmonary metastasis at 146 months. These findings suggest that, beyond local stage, misdiagnosis and inappropriate initial treatment may also compromise long-term outcomes.

ITTC is considered as a radiosensitive tumor due to its histological relationship to thymic carcinoma (25). Most scholars found that the surgical resection of the tumor followed by radiotherapy or combined with chemotherapy provided a better prognosis (13, 26), and Gao also reported that radiotherapy significantly improved survival (13). ITTC is also chemosensitive, with responsiveness reported to cisplatin, doxorubicin, vincristine, cyclophosphamide, carboplatin, and paclitaxel (27). In our series, most patients who received adjuvant radiotherapy or chemoradiotherapy achieved long-term survival without recurrence, whereas one patient who declined adjuvant treatment relapsed within 19 months. Reports of radical radiotherapy alone for advanced ITTC are rare (28). In our study, 4 patients with mediastinal vessel involvement underwent radical chemoradiotherapy, with doses of 60–64 Gy in 3 patients and 70 Gy in 1, consistent with the National Comprehensive Cancer Network (NCCN) guidelines for thymic carcinoma (29). One patient showed poor response and was switched early to anlotinib after multidisciplinary discussion. All 4 patients survived without progression, with follow-up times of 96, 86, 41, and 33 months.

Although multimodality treatment has improved outcomes, preserving physiological function in aggressive tumors may be feasible through neoadjuvant approaches that reduce tumor burden prior to surgery. Chow reported a patient who was not suitable for immediate resection and was successfully managed with neoadjuvant carboplatin and etoposide followed by 66 Gy radiotherapy and surgery, achieving recurrence-free survival 1.8 years after treatment (25). In addition, emerging systemic therapies may further expand treatment options. Anlotinib, a multitarget tyrosine kinase inhibitor, has demonstrated activity in advanced thymic carcinoma (30, 31) and was recently used in life-threatening recurrent ITTC (32). Likewise, pembrolizumab has been approved as a second-line treatment for thymic carcinoma (33), and partial remission has been reported in a metastatic ITTC case (34). Despite these encouraging reports, targeted therapy and immunotherapy have not yet been validated as initial treatment options for locally advanced ITTC, and prospective studies are needed to guide clinical practice. Two cases of ITTC without immunoreactivity to CD5 are alive without recurrence or metastasist with follow-up times of 63, 33 months respectively, one of them received radiotherapy after the operation and another one underwent radical chemoradiotherapy. Can CD5 negativity in patients affect their prognosis? Our research was limited by the small number of patients, perhaps more attention will be paid to the molecular changes of this disease in the future research.

## Conclusions

In this retrospective study of 14 patients with ITTC, the tumors often exhibited invasive features but the prognosis was generally favorable, with a median overall survival of 86 months. Preoperative diagnosis remains challenging, but imaging signs such as an arc interface and hoarseness due to tumor location may aid recognition. CNB with immunohistochemistry is recommended when ITTC is suspected. For locally advanced inoperable ITTC cases, radical chemoradiotherapy is effective, while the role of targeted and immunotherapy requires further exploration.

## Data Availability

The original contributions presented in the study are included in the article/supplementary material. Further inquiries can be directed to the corresponding authors.
